# National surveillance of antimicrobial resistance in Iran using IAMR software: A hospital-based study

**DOI:** 10.1016/j.nmni.2026.101780

**Published:** 2026-05-29

**Authors:** Edris Hoseinzadeh, Hossein Masoumi-Asl

**Affiliations:** a Artificial Intelligence in Environmental Health Research Center (AI-EHRC), Saveh University of Medical Sciences, Saveh, Iran; b Research Center of Pediatric Infectious Diseases, Institute of Immunology and Infectious Diseases, Iran University of Medical Sciences, Tehran, Iran

**Keywords:** Drug resistance, Bacterial, Anti-bacterial agents, Antimicrobial stewardship, Inpatients

## Abstract

**Background:**

Antimicrobial resistance (AMR) is a growing global public health concern that requires standardized and context-specific surveillance systems to inform antimicrobial stewardship and clinical decision-making.

**Objectives:**

This study aimed to evaluate AMR patterns among inpatient and outpatient populations in a tertiary teaching hospital in central Iran and to assess the performance of the Iranian Antimicrobial Resistance Management (IAMR) system in generating stratified surveillance data.

**Methods:**

A cross-sectional study was conducted from September 2023 to July 2024 using routine clinical specimens. Bacterial isolates were identified using standard microbiological methods, and antimicrobial susceptibility testing was performed according to CLSI guidelines. The IAMR system was used for data management, including data cleaning, duplicate removal, and stratification by clinical setting and hospital wards, enabling the generation of antibiograms.

**Results:**

Gram-negative bacteria were predominant in both inpatient and outpatient groups, with *Escherichia coli* accounting for more than 50% of isolates. Resistance rates were higher among inpatients, particularly to third-generation cephalosporins, fluoroquinolones, and trimethoprim–sulfamethoxazole. Intensive care units showed a higher prevalence of multidrug-resistant Klebsiella spp. and *Pseudomonas aeruginosa*. Outpatient isolates exhibited comparatively higher susceptibility rates. The IAMR system facilitated the identification of resistance patterns across different clinical settings.

**Conclusions:**

AMR patterns differed between inpatient and outpatient settings. The use of a structured surveillance system such as IAMR supports the analysis of routine laboratory data and may assist in informing antimicrobial stewardship strategies and alignment with national and international surveillance programs such as GLASS.

## Highlights

• Indigenous IAMR software enabled high-resolution AMR surveillance in Iran.
• Inpatients showed significantly higher resistance than outpatients.
• ICU wards had concentrated multidrug-resistant Klebsiella and Pseudomonas.
• Setting-specific antibiograms improved stewardship-targeted insights.

## Graphical abstract

**Figure:**
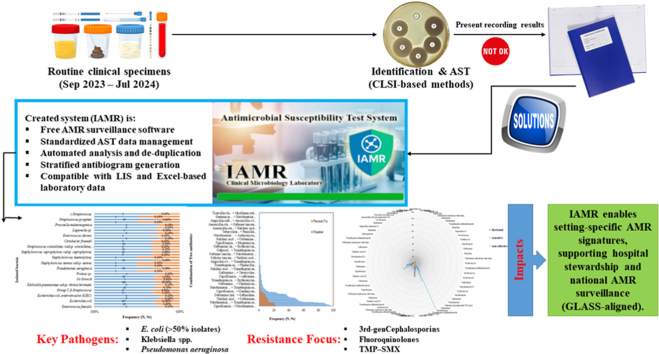

## 1 Introduction

Antimicrobial resistance (AMR) is a major public health concern. Global estimates indicate that approximately 1.27 million deaths were directly attributable to bacterial resistance in 2019, with an additional 4.95 million deaths associated with resistant infections [1,2]. Projections suggest that, without effective interventions, annual mortality related to AMR could reach 10 million by 2050 [3]. In addition to its health impact, AMR imposes a substantial economic burden. In the European Union, resistant infections are associated with more than 33,000 deaths annually and considerable healthcare costs [4,5]. Inappropriate and excessive use of antimicrobial agents is a key contributing factor. Reports from the European Center for Disease Prevention and Control (ECDC) indicate that 30–50% of antibiotic prescriptions in clinical settings may be unnecessary or not aligned with evidence-based guidelines [6,7]. Such prescribing practices increase selective pressure on microorganisms, promoting the emergence and spread of resistant strains [8,9].

In Iran, the antimicrobial resistance (AMR) landscape reflects global trends and poses a substantial challenge to the healthcare system. Multicenter studies have reported high resistance rates among common hospital-associated pathogens, including *Escherichia coli*, *Klebsiella pneumoniae*, *Acinetobacter baumannii*, and *Staphylococcus aureus* [[10], [11], [12], [13]]. The increasing prevalence of multidrug-resistant (MDR) and extensively drug-resistant (XDR) organisms has limited therapeutic options and is associated with longer hospital stays, higher healthcare costs, and increased rates of treatment failure. Antimicrobial susceptibility testing (AST) plays a central role in guiding antibiotic therapy and supporting AMR surveillance [14]. AST data are used to develop institutional antibiograms, monitor temporal trends in resistance, identify resistance mechanisms, and assess the impact of infection control and antimicrobial stewardship programs [[15], [16], [17]]. Accordingly, international guidelines recommend that empirical antimicrobial therapy be guided by local resistance data [17,18]. In response to AMR, the World Health Organization (WHO) introduced the Global Action Plan on Antimicrobial Resistance in 2015 [19,20], which defines five strategic objectives: improving awareness, strengthening surveillance and research, reducing infection incidence, optimizing antimicrobial use, and ensuring sustainable investment. A key component of this strategy is the development of standardized surveillance systems. The WHO subsequently launched the Global Antimicrobial Resistance Surveillance System (GLASS) in 2015 to harmonize data collection and enable comparisons across countries [21]. Following its initial implementation phase (2015–2019), further integration of GLASS principles into national systems has been recommended [22,23]. In Iran, the first National Action Plan to Combat AMR (2016–2021) emphasized the development of information systems and software infrastructure to support surveillance activities [24]. The second National Action Plan has further highlighted the use of locally developed software solutions for systematic AMR data management [[25], [26], [27]]. In this context, the Iranian Antimicrobial Resistance Management (IAMR) software was developed to support the analysis and reporting of AST data. The system is designed to improve data quality, enable standardized monitoring, and facilitate alignment with GLASS requirements.

The present study assessed antimicrobial resistance patterns among inpatient and outpatient populations at Shahid Modarres Hospital in Saveh, central Iran, using antimicrobial susceptibility testing (AST) data collected through the IAMR platform. The findings provide locally relevant evidence that may support antimicrobial stewardship efforts and inform clinical decision-making. Although the study was conducted in a single tertiary-care center, it offers detailed, setting-specific data on antimicrobial resistance based on a structured surveillance system. Such data can contribute to a better understanding of resistance patterns in similar healthcare settings, particularly in low- and middle-income countries where national surveillance systems are still developing.

## 2 Materials and methods

### 2.1 Study setting and design

This descriptive-analytical cross-sectional study was conducted at Shahid Modarres Hospital (Saveh, Iran), a tertiary referral center in Markazi Province. The hospital provides inpatient and outpatient services and includes specialized clinical wards and a central microbiology laboratory for routine microbial culture and antimicrobial susceptibility testing (AST). The study was carried out from September 2023 to July 2024, during which microbiological data and antimicrobial resistance profiles were collected and analyzed.

### 2.2 Study population and inclusion criteria

The study population comprised all inpatients and outpatients referred to the microbiology laboratory with clinical specimens yielding confirmed bacterial growth. This analysis was restricted to bacterial isolates obtained from urine samples, representing cases of urinary tract infections (UTIs) in both community and hospital settings. Inclusion criteria required definitive bacterial identification and a complete antimicrobial susceptibility testing (AST) profile. To avoid overestimation of resistance rates, only the first isolate per patient per specimen type (urine) during the study period was included. Repeated isolates of the same species from the same patient were excluded, in accordance with standard antimicrobial resistance (AMR) surveillance guidelines. A total of 1480 non-duplicate bacterial isolates met the inclusion criteria and were included in the analysis. The final dataset therefore represents unique infection episodes.

### 2.3 Bacterial isolation and identification

Clinical specimens were collected by trained healthcare personnel using aseptic techniques and transported to the laboratory without delay. Samples were cultured on standard media, including Blood Agar, Chocolate Agar, and MacConkey Agar (Merck, Germany), according to specimen type. Plates were incubated aerobically at 37 °C for 24–48 h [28]. Bacterial identification was performed using conventional phenotypic methods, including assessment of colony morphology, Gram staining, and standard biochemical tests (catalase, oxidase, carbohydrate fermentation, indole production, citrate utilization, urease activity, and motility). Specimens with no growth after the incubation period were recorded as culture-negative.

### 2.4 Antimicrobial susceptibility testing (AST)

Antimicrobial susceptibility testing (AST) was conducted using the Kirby–Bauer disk diffusion method on Mueller–Hinton agar in accordance with Clinical and Laboratory Standards Institute (CLSI) guidelines [28]. CLSI breakpoints were applied for all isolates. Plates were incubated at 37 °C for 18–24 h. The antimicrobial agents tested were selected based on local clinical practice and included β-lactams, aminoglycosides, fluoroquinolones, macrolides, glycopeptides, and carbapenems. Results were interpreted as Susceptible (S), Intermediate (I), or Resistant (R) according to CLSI criteria. Multidrug-resistant (MDR) isolates were defined as those non-susceptible to at least one agent in three or more antimicrobial classes.

### 2.5 Data management and processing using IAMR software

The Iranian Antimicrobial Resistance Management (IAMR) software is a web-based surveillance platform developed to standardize the collection, validation, analysis, and reporting of antimicrobial resistance (AMR) data at institutional and national levels. The system integrates microbiology laboratory data, including patient setting (inpatient/outpatient), specimen type, bacterial identification, and antimicrobial susceptibility testing (AST) results. IAMR incorporates automated data cleaning procedures, including removal of duplicate isolates based on patient ID, organism type, and antibiogram similarity, in accordance with international AMR surveillance standards. This deduplication approach ensured that repeated isolates from the same patient did not bias resistance estimates. The software enables stratification of data by hospital wards, specimen types, and clinical settings, allowing generation of both cumulative and unit-specific antibiograms. In addition, IAMR includes analytical modules for calculating resistance frequencies, multidrug resistance rates, and antibiotic coverage indices. The system is designed to be compatible with WHO GLASS requirements, facilitating standardized reporting and potential integration into national and international AMR surveillance networks. Although AMR surveillance ideally integrates detailed clinical data, laboratory-based isolate surveillance remains a widely accepted approach. In this study, a patient-level deduplication strategy (first isolate per patient) was applied, and isolates were stratified by clinical setting (inpatient vs outpatient) to approximate infection origin.

### 2.6 Ethical considerations

The study protocol was approved by the Ethics Committee of Saveh University of Medical Sciences (Reference No: IR. SAVEHUMS.REC.1403.018). Data were analyzed in anonymized form, and no personally identifiable information was used in this study.

## 3 Results and discussion

### 3.1 Bacterial isolates and demographic characteristics

**Fig. 1:**
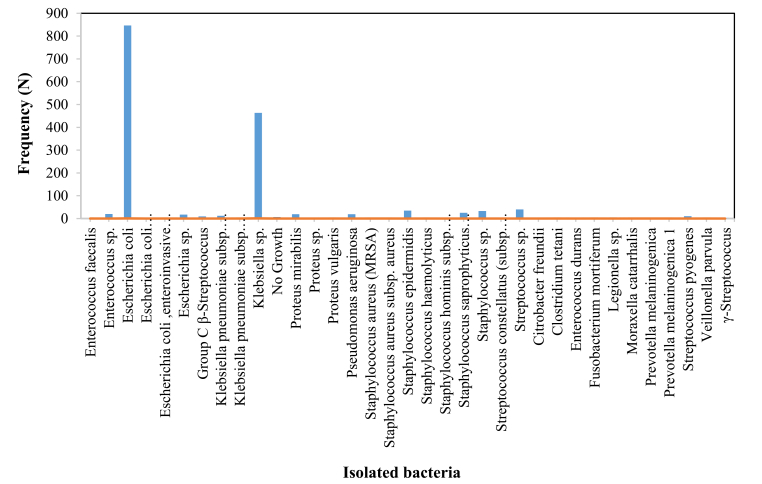
Distribution of bacterial isolates stratified by clinical setting (hospital-acquired and outpatient (community-acquired) isolates, respectively).

In this study, isolates from outpatients were classified as community-acquired, whereas isolates from hospitalized patients were considered hospital-associated. After applying deduplication criteria, 1480 bacterial isolates were included in the final analysis. The distribution of isolates across inpatient and outpatient settings is shown in Fig. 1. Gram-negative bacilli were the predominant pathogens in both groups. *Escherichia coli* was the most frequently isolated organism, accounting for more than 50% of all isolates (details on antimicrobial resistance patterns are provided in Table S1). This finding is consistent with its established role as a leading cause of both community-acquired and healthcare-associated infections, particularly urinary tract infections (UTIs) [29,30]. The similar distribution of *E. coli* in both inpatient and outpatient settings suggests ongoing transmission and circulation of related lineages between community and hospital environments, including resistant strains [31]. *Klebsiella pneumoniae* complex was the second most common group of isolates and was more frequently identified among hospitalized patients (Fig. 1). This higher prevalence in the hospital setting is clinically relevant, as Klebsiella spp. are known to readily acquire resistance mechanisms such as extended-spectrum beta-lactamase (ESBL) production and carbapenemase production [32]. The increased presence of these organisms in inpatient wards likely reflects selective pressure associated with antibiotic use and the persistence of healthcare-associated reservoirs [33,34]. Overall, these results support the need for setting-specific antibiograms to inform empirical treatment, as pathogen distribution and resistance profiles differ between community and hospital settings.

Among non-fermenting Gram-negative bacilli, *Pseudomonas aeruginosa* was detected at a moderate frequency (Fig. 1). Although less common than members of the order Enterobacterales, its presence in both clinical settings is relevant due to its intrinsic resistance mechanisms and ability to acquire multidrug resistance (MDR) during treatment. Gram-positive bacteria showed lower overall isolation rates. *Staphylococcus aureus* and *Staphylococcus saprophyticus* were the most frequently identified Gram-positive species (Fig. 1), while Streptococcus and Enterococcus species were less common. These organisms are recognized as important causes of healthcare- and community-associated infections, respectively. Differences in bacterial distribution were observed between hospitalized and outpatient groups (Fig. 1). Gram-negative opportunistic pathogens were more frequent among inpatients, whereas Gram-positive community-associated organisms were relatively more common in outpatients. These patterns suggest an association between healthcare exposure, antimicrobial use, and bacterial distribution [35].

The predominance of Gram-negative organisms, particularly *Escherichia coli*, in both cohorts is consistent with reports from the Global Antimicrobial Resistance Surveillance System (GLASS) and regional studies in the Middle East [36,37]. This may reflect the combined burden of community- and hospital-acquired infections and possible transmission of Enterobacterales across healthcare and community settings. In addition, the higher proportion of Klebsiella spp. among hospitalized patients, especially in high-acuity units, was identified through IAMR-based stratification, indicating stronger selective pressure in hospital environments and supporting the value of structured digital resistance surveillance systems in clinical practice.

### 3.2 Antibiotic resistance patterns in hospitalized and outpatient populations

**Fig. 2:**
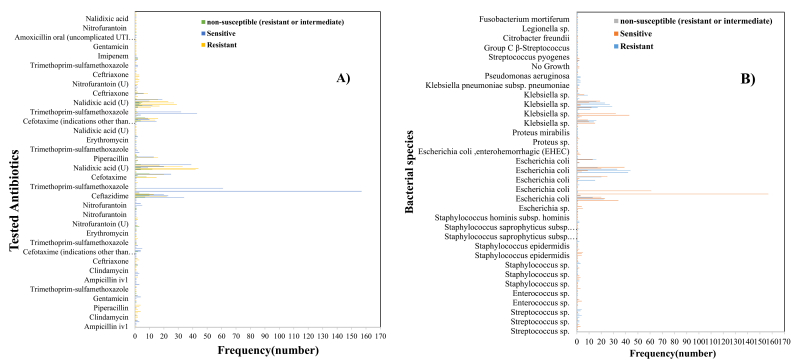
Antimicrobial resistance patterns among clinical isolates. (A) Frequency (number) of resistant isolates for each antibiotic tested, based on CLSI-defined resistance criteria. (B) Frequency (number) of resistant isolates stratified by bacterial species.

The antimicrobial resistance data are presented in two complementary formats (Fig. 2). Resistance rates for individual antibiotics are expressed as the proportion of resistant isolates among all tested samples (Fig. 2A), while the absolute number of resistant isolates is shown by bacterial species to describe the distribution of resistance across pathogens (Fig. 2B). This approach enables assessment of antibiotic resistance levels and comparison of major resistant organisms within the study population. As shown in Fig. 2, resistance patterns differed between Gram-negative and Gram-positive bacteria and across clinical settings. Among Gram-negative pathogens, *Escherichia coli* showed high resistance to ciprofloxacin (72.4%), nalidixic acid (77.2%), and trimethoprim–sulfamethoxazole (62.3%), with resistance to third-generation cephalosporins ranging from approximately 30% to 50%. Klebsiella spp. Exhibited higher resistance levels, with over 70% of isolates resistant to cefotaxime, ciprofloxacin, and nalidixic acid. *Pseudomonas aeruginosa* showed resistance to all tested antibiotics (100%), indicating severely limited therapeutic options. Gram-positive organisms generally demonstrated lower resistance rates, although exceptions were observed. Streptococcus spp. Showed complete resistance (100%) to ampicillin, erythromycin, clindamycin, tetracycline, and ciprofloxacin. Within the Staphylococcus genus, *S. epidermidis* and other staphylococci showed variable susceptibility, with some isolates exhibiting complete resistance to clindamycin and ciprofloxacin. Enterococcus spp. remained largely susceptible to nitrofurantoin and penicillin, despite observed resistance to third-generation cephalosporins. Comparative analysis indicated higher resistance rates among hospitalized patients, particularly for third-generation cephalosporins and fluoroquinolones. This pattern is consistent with previous reports from Iran and the broader Middle East, and may be associated with longer hospital stays, prior antibiotic exposure, and increased selective pressure in inpatient settings [37,38].

### 3.3 Antibiotic Coverage Percentages in hospitalized and outpatient isolates

**Fig. 3:**
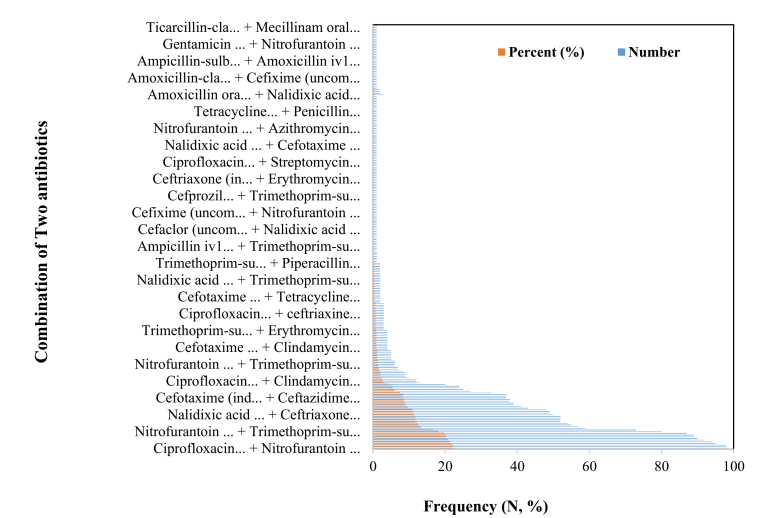
Antibiotic Coverage Percentages in this study.

Fig. 3 shows the cumulative coverage percentages of the evaluated antimicrobials against clinical isolates. The results indicate variability in coverage among antibiotic classes, reflecting differences in susceptibility patterns of the tested pathogens. A limited number of antibiotics demonstrated high coverage, whereas several commonly used agents showed reduced effectiveness. This suggests a decrease in the reliability of some first-line empirical treatments in this clinical setting. The use of IAMR-generated coverage-based summaries provides additional information for evaluating antimicrobial performance and may support more informed empirical treatment decisions. Such coverage-based analysis is not always included in conventional surveillance systems, which often focus primarily on resistance prevalence rather than clinical coverage metrics.

The observed distribution may be associated with factors such as prior antimicrobial use, prolonged hospitalization, and the presence of resistant sub-populations in the hospital environment. These findings highlight the need for data-driven antimicrobial stewardship to help preserve the effectiveness of available antimicrobial agents.

### 3.4 Patterns of resistant bacteria across clinical wards

**Fig. 4:**
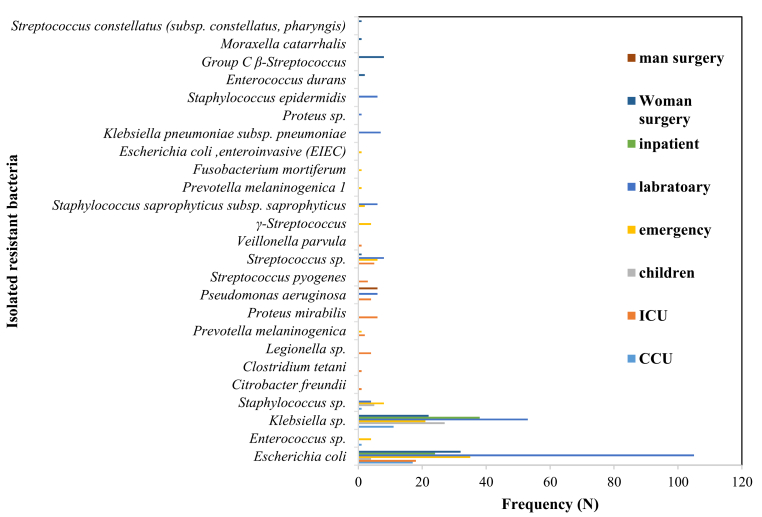
Ward-specific distribution of antimicrobial-resistant bacterial isolates.

The coverage patterns presented in Fig. 4 highlight the importance of using local antibiogram data in clinical decision-making. Inadequate antimicrobial coverage may increase the likelihood of treatment failure. Regular evaluation of coverage rates can support more appropriate empirical therapy selection and contribute to antimicrobial stewardship programs in hospital settings [39,40]. The distribution of resistant isolates varied across hospital wards and outpatient-related settings. *Escherichia coli* was the most frequently isolated resistant organism in most settings, particularly among inpatients, emergency department cases, and laboratory referrals. This is consistent with its established role in both community-acquired and healthcare-associated infections [41]. Klebsiella spp. were the second most common resistant isolates, mainly identified in hospitalized patients, especially in intensive care and coronary care units (Fig. 4). Their higher frequency in these wards may be associated with longer hospital stays, invasive procedures, and exposure to broad-spectrum antibiotics [42]. Resistant Staphylococcus spp. were detected across multiple clinical settings, including surgical wards, inpatient units, and emergency departments (Fig. 4). Their presence in these areas is consistent with their known involvement in skin, soft tissue, and device-related infections, and supports the need for continued infection control measures alongside stewardship activities [43,44]. Non-fermenting Gram-negative bacilli, particularly *Pseudomonas aeruginosa*, were less frequently isolated but were mainly found in intensive care units (Fig. 4), consistent with their association with severe infections in critically ill patients and intrinsic resistance mechanisms. Other organisms, including Enterococcus spp., Proteus spp., Streptococcus spp., and Citrobacter spp., were detected at lower frequencies across different wards, contributing to the overall resistance profile of the hospital. Ward-level analysis showed that multidrug-resistant Klebsiella spp. and *Pseudomonas aeruginosa* were more concentrated in intensive care units. These findings support the use of unit-specific antibiograms rather than relying solely on hospital-wide aggregated data. The availability of detailed ward-level resistance data through IAMR can improve the precision of antimicrobial stewardship interventions by aligning them with local resistance patterns.

Antimicrobial resistance (AMR) showed variation across hospital departments, with *Escherichia coli* and Klebsiella spp. as the most frequently isolated resistant pathogens. In the intensive care unit (ICU), resistance was observed in *E. coli*, *Proteus mirabilis*, and *Pseudomonas aeruginosa* against third-generation cephalosporins, fluoroquinolones, and commonly used urinary antibiotics, including nalidixic acid, nitrofurantoin, and trimethoprim–sulfamethoxazole. In the coronary care unit (CCU) and inpatient wards, resistance was mainly identified in *E. coli* and Klebsiella spp. to ceftriaxone, ceftazidime, ciprofloxacin, and trimethoprim–sulfamethoxazole. In pediatric and female surgical wards, resistance patterns were broadly similar, with additional isolation of Streptococcus spp. and Group C β-hemolytic Streptococcus in surgical settings. The emergency department showed a mixed distribution of resistant Gram-negative and Gram-positive organisms. In the male surgical ward, *P. aeruginosa* was the predominant resistant organism across several antibiotic classes (further details on ward-specific distribution of antimicrobial-resistant bacteria are provided in Table S2). Overall, resistance was most frequently associated with third-generation cephalosporins, fluoroquinolones, and trimethoprim–sulfamethoxazole, indicating substantial selective pressure associated with these agents across departments. These findings support the consideration of department-specific antimicrobial stewardship strategies to optimize antimicrobial use and address AMR in the clinical setting.

## 4 Conclusion

This study evaluated the Iranian Antimicrobial Resistance Management (IAMR) software as a locally developed surveillance tool for monitoring antimicrobial resistance (AMR) patterns in a tertiary hospital in Iran. Gram-negative bacteria, particularly *Escherichia coli* and *Klebsiella pneumoniae*, were the most frequently isolated pathogens in both inpatient and outpatient settings. Higher resistance rates were observed among hospitalized patients, especially to third-generation cephalosporins and fluoroquinolones. Ward-level analysis indicated a higher prevalence of multidrug-resistant organisms, notably Klebsiella spp. and *Pseudomonas aeruginosa*, in intensive care units. The IAMR system enabled automated removal of duplicate records, integration of laboratory information system data, and stratification of results by clinical setting and hospital ward. It also facilitated the generation of hospital-level and ward-specific antibiograms, as well as estimates of antibiotic coverage to support empirical therapy decisions. In addition, the system was designed in accordance with the GLASS framework, supporting compatibility with national and international AMR surveillance standards. In conclusion, the system provides a structured approach for organizing routine microbiological data into surveillance outputs that can support AMR monitoring and clinical decision-making.

### 4.1 Limitations of the study

Several limitations should be acknowledged. The single-center design limits the generalizability of the findings. In addition, the use of disk diffusion methods may not detect all underlying resistance mechanisms. The lack of detailed clinical data, including prior antibiotic exposure, comorbidities, and patient outcomes, restricts assessment of the clinical implications of resistance patterns. Moreover, the cross-sectional design does not allow evaluation of temporal trends in antimicrobial resistance. Future multicenter studies incorporating clinical and molecular data are needed to validate and extend these findings. The IAMR software may be considered as a supportive component in antimicrobial stewardship and surveillance systems, although further evaluation is required to confirm its utility across different settings.

## Ethical approval

The study was conducted in accordance with ethical standards. Ethical approval was obtained from the Ethics Committee of Saveh University of Medical Sciences (approval code: IR. SAVEHUMS.REC.1403.018). Written informed consent was obtained from all participants. Participant confidentiality and data privacy were maintained throughout the study in accordance with applicable data protection regulations.

## Data availability statement

The datasets supporting the conclusions of this study are available from the corresponding author upon reasonable request.

## AI tools usage statement

During the preparation of this manuscript, the authors used ChatGPT solely as a language-editing tool to improve grammar, spelling, and clarity. The authors critically reviewed and revised all content and assume full responsibility for the final version of the manuscript.

## Funding

The authors acknowledge financial support from the 10.13039/501100012155National Institute for Medical Research Development (10.13039/501100012155NIMAD) under grant number #4031096, which supported this research.

## CRediT authorship contribution statement

**Edris Hoseinzadeh:** Conceptualization, Data curation, Formal analysis, Investigation, Methodology, Software, Supervision, Validation, Visualization, Writing – original draft, Writing – review & editing. **Hossein Masoumi-Asl:** Data curation, Formal analysis, Investigation, Methodology, Software, Visualization, Writing – original draft.

## Declaration of competing interest

The authors declare that they have no known competing financial interests or personal relationships that could have appeared to influence the work reported in this paper. All experiments, analyses, and interpretations were conducted independently, without any financial, commercial, or personal conflict of interest that might bias the outcomes of this research.
