# Supplementary material for: National surveillance of antimicrobial resistance in Iran using IAMR software: A hospital-based study
Source: New Microbes New Infect. 2026 May 29;72:101780. doi: 10.1016/j.nmni.2026.101780 (PMC13251500; doi:10.1016/j.nmni.2026.101780)
Supplement: Multimedia component 1 [file mmc1.docx]

**Table S1. Antibiotic resistance patterns**

| **Microorganisms** | **Antibiotics** | **Resistance** | | **Sensitive** | | **Non-effective** | |
| --- | --- | --- | --- | --- | --- | --- | --- |
|  |  | **(%)** | **N** | **(%)** | **N** | **(%)** | **N** |
| Streptococcus sp. | Ampicillin iv1 | 100 | 1 | 0 | 0 | 0 | 0 |
| Streptococcus sp. | Cefotaxime (indications other than meningitis) | 0 | 0 | 100 | 1 | 0 | 0 |
| Streptococcus sp. | Gentamicin | 0 | 0 | 100 | 1 | 0 | 0 |
| Streptococcus sp. | Nitrofurantoin | 0 | 0 | 100 | 3 | 0 | 0 |
| Streptococcus sp. | Trimethoprim-sulfamethoxazole | 33.33 | 1 | 66.67 | 2 | 0 | 0 |
| Streptococcus sp. | Erythromycin | 100 | 1 | 0 | 0 | 0 | 0 |
| Streptococcus sp. | Clindamycin | 100 | 2 | 0 | 0 | 0 | 0 |
| Streptococcus sp. | Ampicillin | 66.67 | 2 | 33.33 | 1 | 0 | 0 |
| Streptococcus sp. | Cefotaxime | 100 | 1 | 0 | 0 | 0 | 0 |
| Streptococcus sp. | Tetracycline | 100 | 2 | 0 | 0 | 0 | 0 |
| Streptococcus sp. | Ciprofloxacin | 100 | 4 | 0 | 0 | 0 | 0 |
| Streptococcus sp. | Trimethoprim-sulfamethoxazole | 100 | 2 | 0 | 0 | 0 | 0 |
| Streptococcus sp. | Piperacillin | 50 | 1 | 50 | 1 | 0 | 0 |
| Streptococcus sp. | Erythromycin | 100 | 3 | 0 | 0 | 0 | 0 |
| Streptococcus sp. | Clindamycin | 100 | 4 | 0 | 0 | 0 | 0 |
| Group C β-Streptococcus | Cefotaxime (indications other than meningitis) | 0 | 0 | 0 | 0 | 100 | 1 |
| Enterococcus sp. | Cefotaxime (indications other than meningitis) | 0 | 0 | 100 | 1 | 0 | 0 |
| Enterococcus sp. | Ceftriaxone (indications other than meningitis) | 100 | 1 | 0 | 0 | 0 | 0 |
| Enterococcus sp. | Gentamicin | 0 | 0 | 100 | 1 | 0 | 0 |
| Enterococcus sp. | Nitrofurantoin | 0 | 0 | 100 | 4 | 0 | 0 |
| Enterococcus sp. | Trimethoprim-sulfamethoxazole | 33.33 | 1 | 66.67 | 2 | 0 | 0 |
| Enterococcus sp. | Ceftazidime | 0 | 0 | 0 | 0 | 100 | 1 |
| Enterococcus sp. | Ciprofloxacin | 0 | 0 | 0 | 0 | 100 | 1 |
| Enterococcus sp. | Nalidixic acid (U) | 100 | 1 | 0 | 0 | 0 | 0 |
| Enterococcus sp. | Trimethoprim-sulfamethoxazole | 100 | 1 | 0 | 0 | 0 | 0 |
| Enterococcus sp. | Nitrofurantoin (U) | 0 | 0 | 100 | 1 | 0 | 0 |
| Enterococcus sp. | Ceftriaxone | 100 | 1 | 0 | 0 | 0 | 0 |
| Enterococcus sp. | Penicillin | 0 | 0 | 100 | 3 | 0 | 0 |
| Enterococcus faecalis | Nitrofurantoin | 0 | 0 | 100 | 1 | 0 | 0 |
| Enterococcus faecalis | Trimethoprim-sulfamethoxazole | 0 | 0 | 100 | 1 | 0 | 0 |
| Staphylococcus sp. | Ampicillin iv1 | 0 | 0 | 100 | 1 | 0 | 0 |
| Staphylococcus sp. | Cefotaxime (indications other than meningitis) | 0 | 0 | 100 | 1 | 0 | 0 |
| Staphylococcus sp. | Ceftriaxone (indications other than meningitis) | 100 | 2 | 0 | 0 | 0 | 0 |
| Staphylococcus sp. | Nitrofurantoin | 0 | 0 | 100 | 2 | 0 | 0 |
| Staphylococcus sp. | Trimethoprim-sulfamethoxazole | 0 | 0 | 100 | 3 | 0 | 0 |
| Staphylococcus sp. | Erythromycin | 0 | 0 | 100 | 2 | 0 | 0 |
| Staphylococcus sp. | Clindamycin | 100 | 1 | 0 | 0 | 0 | 0 |
| Staphylococcus sp. | Cefotaxime | 100 | 1 | 0 | 0 | 0 | 0 |
| Staphylococcus sp. | Tetracycline | 100 | 1 | 0 | 0 | 0 | 0 |
| Staphylococcus sp. | Ciprofloxacin | 33.33 | 1 | 0 | 0 | 66.67 | 2 |
| Staphylococcus sp. | Trimethoprim-sulfamethoxazole | 33.33 | 1 | 66.67 | 2 | 0 | 0 |
| Staphylococcus sp. | Nitrofurantoin (U) | 100 | 1 | 0 | 0 | 0 | 0 |
| Staphylococcus sp. | Ceftriaxone | 100 | 1 | 0 | 0 | 0 | 0 |
| Staphylococcus sp. | Erythromycin | 100 | 3 | 0 | 0 | 0 | 0 |
| Staphylococcus sp. | Clindamycin | 66.67 | 2 | 33.33 | 1 | 0 | 0 |
| Staphylococcus sp. | Penicillin | 0 | 0 | 100 | 1 | 0 | 0 |
| Staphylococcus aureus subsp. aureus | Trimethoprim-sulfamethoxazole | 0 | 0 | 100 | 1 | 0 | 0 |
| Staphylococcus aureus (MRSA) | Trimethoprim-sulfamethoxazole | 0 | 0 | 100 | 1 | 0 | 0 |
| Staphylococcus epidermidis | Cefotaxime (indications other than meningitis) | 0 | 0 | 66.67 | 4 | 33.33 | 2 |
| Staphylococcus epidermidis | Nitrofurantoin | 0 | 0 | 100 | 4 | 0 | 0 |
| Staphylococcus epidermidis | Trimethoprim-sulfamethoxazole | 0 | 0 | 100 | 5 | 0 | 0 |
| Staphylococcus epidermidis | Erythromycin | 0 | 0 | 100 | 1 | 0 | 0 |
| Staphylococcus epidermidis | Clindamycin | 0 | 0 | 100 | 2 | 0 | 0 |
| Staphylococcus epidermidis | Ciprofloxacin | 50 | 1 | 0 | 0 | 50 | 1 |
| Staphylococcus epidermidis | Trimethoprim-sulfamethoxazole | 50 | 1 | 50 | 1 | 0 | 0 |
| Staphylococcus epidermidis | Nitrofurantoin (U) | 0 | 0 | 100 | 1 | 0 | 0 |
| Staphylococcus epidermidis | Ampicillin | 100 | 1 | 0 | 0 | 0 | 0 |
| Staphylococcus epidermidis | Erythromycin | 100 | 2 | 0 | 0 | 0 | 0 |
| Staphylococcus epidermidis | Clindamycin | 50 | 1 | 0 | 0 | 50 | 1 |
| Staphylococcus saprophyticus subsp. saprophyticus | Nitrofurantoin | 0 | 0 | 100 | 1 | 0 | 0 |
| Staphylococcus saprophyticus subsp. saprophyticus | Erythromycin | 100 | 1 | 0 | 0 | 0 | 0 |
| Staphylococcus saprophyticus subsp. saprophyticus | Ampicillin | 0 | 0 | 100 | 1 | 0 | 0 |
| Staphylococcus saprophyticus subsp. saprophyticus | Cefotaxime | 33.33 | 1 | 33.33 | 1 | 33.33 | 1 |
| Staphylococcus saprophyticus subsp. saprophyticus | Ceftazidime | 0 | 0 | 0 | 0 | 100 | 1 |
| Staphylococcus saprophyticus subsp. saprophyticus | Ciprofloxacin | 0 | 0 | 25 | 1 | 75 | 3 |
| Staphylococcus saprophyticus subsp. saprophyticus | Trimethoprim-sulfamethoxazole | 66.67 | 2 | 33.33 | 1 | 0 | 0 |
| Staphylococcus saprophyticus subsp. saprophyticus | Nitrofurantoin (U) | 0 | 0 | 100 | 1 | 0 | 0 |
| Staphylococcus saprophyticus subsp. saprophyticus | Piperacillin | 0 | 0 | 100 | 1 | 0 | 0 |
| Staphylococcus saprophyticus subsp. saprophyticus | Ceftriaxone | 0 | 0 | 100 | 1 | 0 | 0 |
| Staphylococcus saprophyticus subsp. saprophyticus | Erythromycin | 66.67 | 2 | 0 | 0 | 33.33 | 1 |
| Staphylococcus saprophyticus subsp. saprophyticus | Clindamycin | 100 | 2 | 0 | 0 | 0 | 0 |
| Staphylococcus saprophyticus subsp. saprophyticus | Penicillin | 0 | 0 | 100 | 1 | 0 | 0 |
| Staphylococcus hominis subsp. hominis | Nitrofurantoin | 0 | 0 | 100 | 1 | 0 | 0 |
| Staphylococcus hominis subsp. hominis | Clindamycin | 0 | 0 | 100 | 1 | 0 | 0 |
| Staphylococcus hominis subsp. hominis | Penicillin | 0 | 0 | 100 | 1 | 0 | 0 |
| Escherichia sp. | Ceftazidime | 0 | 0 | 50 | 1 | 50 | 1 |
| Escherichia sp. | Ceftriaxone (indications other than meningitis) | 33.33 | 1 | 33.33 | 1 | 33.33 | 1 |
| Escherichia sp. | Gentamicin | 0 | 0 | 100 | 1 | 0 | 0 |
| Escherichia sp. | Nitrofurantoin | 0 | 0 | 100 | 5 | 0 | 0 |
| Escherichia sp. | Trimethoprim-sulfamethoxazole | 0 | 0 | 100 | 4 | 0 | 0 |
| Escherichia coli | Ampicillin iv1 | 0 | 0 | 100 | 1 | 0 | 0 |
| Escherichia coli | Cefalexin (uncomplicated UTI only) | 0 | 0 | 100 | 1 | 0 | 0 |
| Escherichia coli | Cefixime (uncomplicated UTI only) | 0 | 0 | 100 | 1 | 0 | 0 |
| Escherichia coli | Cefotaxime (indications other than meningitis) | 2.38 | 1 | 80.95 | 34 | 16.67 | 7 |
| Escherichia coli | Ceftazidime | 40 | 22 | 41.82 | 23 | 18.18 | 10 |
| Escherichia coli | Ceftriaxone (indications other than meningitis) | 30.23 | 13 | 46.51 | 20 | 23.26 | 10 |
| Escherichia coli | Gentamicin | 50 | 3 | 50 | 3 | 0 | 0 |
| Escherichia coli | Nitrofurantoin | 0 | 0 | 100 | 157 | 0 | 0 |
| Escherichia coli | Nitroxoline E. coli | 100 | 1 | 0 | 0 | 0 | 0 |
| Escherichia coli | Trimethoprim-sulfamethoxazole | 1.61 | 1 | 98.39 | 61 | 0 | 0 |
| Escherichia coli | Trimethoprim-sulfamethoxazole | 0 | 0 | 0 | 0 | 100 | 1 |
| Escherichia coli | Erythromycin | 0 | 0 | 100 | 1 | 0 | 0 |
| Escherichia coli | Nitrofurantoin | 0 | 0 | 100 | 1 | 0 | 0 |
| Escherichia coli | Nalidixic acid | 100 | 2 | 0 | 0 | 0 | 0 |
| Escherichia coli | Streptomycin | 0 | 0 | 100 | 1 | 0 | 0 |
| Escherichia coli | Amoxicillin-clavulanate | 0 | 0 | 100 | 1 | 0 | 0 |
| Escherichia coli | Cefotaxime | 62.5 | 15 | 4.17 | 1 | 33.33 | 8 |
| Escherichia coli | ceftriaxine | 0 | 0 | 66.67 | 2 | 33.33 | 1 |
| Escherichia coli | Ceftazidime | 36.36 | 20 | 45.45 | 25 | 18.18 | 10 |
| Escherichia coli | Cefprozil | 0 | 0 | 0 | 0 | 100 | 1 |
| Escherichia coli | Tetracycline | 100 | 1 | 0 | 0 | 0 | 0 |
| Escherichia coli | Ciprofloxacin | 72.41 | 42 | 6.9 | 4 | 20.69 | 12 |
| Escherichia coli | Nalidixic acid (U) | 77.19 | 44 | 15.79 | 9 | 7.02 | 4 |
| Escherichia coli | Trimethoprim-sulfamethoxazole | 62.26 | 33 | 37.74 | 20 | 0 | 0 |
| Escherichia coli | Nitrofurantoin (U) | 28.33 | 17 | 65 | 39 | 6.67 | 4 |
| Escherichia coli | Azithromycin | 0 | 0 | 0 | 0 | 100 | 1 |
| Escherichia coli | Ciprofloxacin | 0 | 0 | 0 | 0 | 100 | 1 |
| Escherichia coli | Trimethoprim-sulfamethoxazole | 50 | 2 | 50 | 2 | 0 | 0 |
| Escherichia coli | Piperacillin | 0 | 0 | 0 | 0 | 100 | 1 |
| Escherichia coli | Ceftriaxone | 51.61 | 16 | 41.94 | 13 | 6.45 | 2 |
| Escherichia coli | Erythromycin | 50 | 1 | 0 | 0 | 50 | 1 |
| Escherichia coli | Clindamycin | 100 | 1 | 0 | 0 | 0 | 0 |
| Escherichia coli | Penicillin | 0 | 0 | 100 | 3 | 0 | 0 |
| Escherichia coli ,enterohemorrhagic (EHEC) | Nitrofurantoin | 0 | 0 | 100 | 2 | 0 | 0 |
| Escherichia coli ,enterohemorrhagic (EHEC) | Trimethoprim-sulfamethoxazole | 0 | 0 | 100 | 1 | 0 | 0 |
| Escherichia coli ,enteroinvasive (EIEC) | Ceftriaxone (indications other than meningitis) | 100 | 1 | 0 | 0 | 0 | 0 |
| Escherichia coli ,enteroinvasive (EIEC) | Nitrofurantoin | 0 | 0 | 100 | 1 | 0 | 0 |
| Proteus sp. | Cefotaxime (indications other than meningitis) | 0 | 0 | 100 | 1 | 0 | 0 |
| Proteus sp. | Ceftriaxone (indications other than meningitis) | 0 | 0 | 0 | 0 | 100 | 1 |
| Proteus sp. | Nitrofurantoin | 0 | 0 | 100 | 1 | 0 | 0 |
| Proteus sp. | Erythromycin | 100 | 1 | 0 | 0 | 0 | 0 |
| Proteus mirabilis | Cefalexin (uncomplicated UTI only) | 0 | 0 | 100 | 1 | 0 | 0 |
| Proteus mirabilis | Ceftriaxone (indications other than meningitis) | 0 | 0 | 66.67 | 2 | 33.33 | 1 |
| Proteus mirabilis | Trimethoprim-sulfamethoxazole | 0 | 0 | 100 | 1 | 0 | 0 |
| Proteus mirabilis | Ceftazidime | 100 | 1 | 0 | 0 | 0 | 0 |
| Proteus mirabilis | Ciprofloxacin | 100 | 1 | 0 | 0 | 0 | 0 |
| Proteus mirabilis | Nalidixic acid (U) | 100 | 1 | 0 | 0 | 0 | 0 |
| Proteus mirabilis | Trimethoprim-sulfamethoxazole | 100 | 1 | 0 | 0 | 0 | 0 |
| Proteus mirabilis | Nitrofurantoin (U) | 100 | 1 | 0 | 0 | 0 | 0 |
| Proteus mirabilis | Ceftriaxone | 100 | 1 | 0 | 0 | 0 | 0 |
| Proteus vulgaris | Nitrofurantoin | 0 | 0 | 100 | 1 | 0 | 0 |
| Klebsiella sp. | Temocillin (infections originating from the urinary tract), E. coli, Klebsiella spp. (except K. aerogenes) and P. mirabilis | 0 | 0 | 0 | 0 | 100 | 1 |
| Klebsiella sp. | Cefotaxime (indications other than meningitis) | 5 | 1 | 75 | 15 | 20 | 4 |
| Klebsiella sp. | Ceftazidime | 53.85 | 14 | 38.46 | 10 | 7.69 | 2 |
| Klebsiella sp. | Ceftriaxone (indications other than meningitis) | 50 | 16 | 28.12 | 9 | 21.88 | 7 |
| Klebsiella sp. | Ciprofloxacin | 0 | 0 | 100 | 1 | 0 | 0 |
| Klebsiella sp. | Gentamicin | 33.33 | 2 | 66.67 | 4 | 0 | 0 |
| Klebsiella sp. | Nitrofurantoin | 2.27 | 1 | 97.73 | 43 | 0 | 0 |
| Klebsiella sp. | Trimethoprim-sulfamethoxazole | 5.71 | 2 | 91.43 | 32 | 2.86 | 1 |
| Klebsiella sp. | Cefazolin | 100 | 1 | 0 | 0 | 0 | 0 |
| Klebsiella sp. | Ceftaroline | 100 | 1 | 0 | 0 | 0 | 0 |
| Klebsiella sp. | Cefotaxime | 73.33 | 11 | 13.33 | 2 | 13.33 | 2 |
| Klebsiella sp. | Ceftazidime | 50 | 17 | 35.29 | 12 | 14.71 | 5 |
| Klebsiella sp. | Ciprofloxacin | 80.56 | 29 | 8.33 | 3 | 11.11 | 4 |
| Klebsiella sp. | Nalidixic acid (U) | 77.14 | 27 | 8.57 | 3 | 14.29 | 5 |
| Klebsiella sp. | Trimethoprim-sulfamethoxazole | 69.7 | 23 | 30.3 | 10 | 0 | 0 |
| Klebsiella sp. | Nitrofurantoin (U) | 44.44 | 16 | 52.78 | 19 | 2.78 | 1 |
| Klebsiella sp. | Ciprofloxacin | 100 | 1 | 0 | 0 | 0 | 0 |
| Klebsiella sp. | Trimethoprim-sulfamethoxazole | 100 | 2 | 0 | 0 | 0 | 0 |
| Klebsiella sp. | Cefotaxime | 0 | 0 | 100 | 1 | 0 | 0 |
| Klebsiella sp. | Ceftriaxone | 56.25 | 9 | 37.5 | 6 | 6.25 | 1 |
| Klebsiella pneumoniae subsp. pneumoniae | Cefotaxime | 100 | 2 | 0 | 0 | 0 | 0 |
| Klebsiella pneumoniae subsp. pneumoniae | Ceftazidime | 50 | 1 | 50 | 1 | 0 | 0 |
| Klebsiella pneumoniae subsp. pneumoniae | Ciprofloxacin | 50 | 1 | 50 | 1 | 0 | 0 |
| Klebsiella pneumoniae subsp. pneumoniae | Nalidixic acid (U) | 0 | 0 | 100 | 2 | 0 | 0 |
| Klebsiella pneumoniae subsp. pneumoniae | Trimethoprim-sulfamethoxazole | 50 | 1 | 50 | 1 | 0 | 0 |
| Klebsiella pneumoniae subsp. pneumoniae | Nitrofurantoin (U) | 100 | 2 | 0 | 0 | 0 | 0 |
| Pseudomonas aeruginosa | Ceftazidime | 100 | 2 | 0 | 0 | 0 | 0 |
| Pseudomonas aeruginosa | Ciprofloxacin | 100 | 3 | 0 | 0 | 0 | 0 |
| Pseudomonas aeruginosa | Nalidixic acid (U) | 100 | 3 | 0 | 0 | 0 | 0 |
| Pseudomonas aeruginosa | Trimethoprim-sulfamethoxazole | 100 | 2 | 0 | 0 | 0 | 0 |
| Pseudomonas aeruginosa | Nitrofurantoin (U) | 100 | 3 | 0 | 0 | 0 | 0 |
| Pseudomonas aeruginosa | Ceftriaxone | 100 | 3 | 0 | 0 | 0 | 0 |
| Streptococcus constellatus (subsp. constellatus, pharyngis) | Ceftriaxone (indications other than meningitis) | 100 | 1 | 0 | 0 | 0 | 0 |
| Streptococcus constellatus (subsp. constellatus, pharyngis) | Penicillin | 0 | 0 | 100 | 1 | 0 | 0 |
| No Growth | Cefotaxime (indications other than meningitis) | 100 | 1 | 0 | 0 | 0 | 0 |
| No Growth | Gentamicin | 100 | 1 | 0 | 0 | 0 | 0 |
| No Growth | Nitrofurantoin | 0 | 0 | 100 | 2 | 0 | 0 |
| No Growth | Trimethoprim-sulfamethoxazole | 0 | 0 | 100 | 2 | 0 | 0 |
| γ-Streptococcus | Ticarcillin-clavulanic acid | 100 | 1 | 0 | 0 | 0 | 0 |
| γ-Streptococcus | Gentamicin | 100 | 1 | 0 | 0 | 0 | 0 |
| γ-Streptococcus | Metronidazole | 0 | 0 | 100 | 1 | 0 | 0 |
| Streptococcus pyogenes | Amoxicillin-clavulanic acid iv1 | 33.33 | 2 | 33.33 | 2 | 33.33 | 2 |
| Streptococcus pyogenes | Cefixime (uncomplicated UTI only) | 33.33 | 1 | 66.67 | 2 | 0 | 0 |
| Streptococcus pyogenes | Imipenem | 0 | 0 | 100 | 1 | 0 | 0 |
| Group C β-Streptococcus | Ampicillin iv1 | 100 | 1 | 0 | 0 | 0 | 0 |
| Group C β-Streptococcus | Ampicillin oral (uncomplicated UTI only)1 | 100 | 1 | 0 | 0 | 0 | 0 |
| Group C β-Streptococcus | Ampicillin-sulbactam iv1 | 100 | 1 | 0 | 0 | 0 | 0 |
| Group C β-Streptococcus | Amoxicillin iv1 | 100 | 1 | 0 | 0 | 0 | 0 |
| Group C β-Streptococcus | Ceftibuten (infections originating from the urinary tract) | 100 | 1 | 0 | 0 | 0 | 0 |
| Group C β-Streptococcus | Gentamicin | 100 | 1 | 0 | 0 | 0 | 0 |
| Group C β-Streptococcus | Doripenem | 100 | 1 | 0 | 0 | 0 | 0 |
| Group C β-Streptococcus | Azithromycin | 100 | 1 | 0 | 0 | 0 | 0 |
| Enterococcus durans | Levofloxacin | 0 | 0 | 100 | 1 | 0 | 0 |
| Enterococcus durans | Minocycline | 100 | 1 | 0 | 0 | 0 | 0 |
| Enterococcus durans | Doxycycline | 100 | 1 | 0 | 0 | 0 | 0 |
| Citrobacter freundii | Amoxicillin oral (uncomplicated UTI only)1 | 0 | 0 | 100 | 1 | 0 | 0 |
| Citrobacter freundii | Amoxicillin-clavulanic acid oral (infections originating from the urinary tract)1 | 0 | 0 | 100 | 1 | 0 | 0 |
| Citrobacter freundii | Nalidixic acid | 100 | 1 | 0 | 0 | 0 | 0 |
| Legionella sp. | Ticarcillin-clavulanic acid | 0 | 0 | 0 | 0 | 100 | 1 |
| Legionella sp. | Mecillinam oral (pivmecillinam) (uncomplicated UTI only), E. coli, Citrobacter spp., Klebsiella spp., Raoultella spp., Enterobacter spp. and P. mirabilis | 100 | 1 | 0 | 0 | 0 | 0 |
| Legionella sp. | Gentamicin | 100 | 1 | 0 | 0 | 0 | 0 |
| Legionella sp. | Nitrofurantoin | 100 | 1 | 0 | 0 | 0 | 0 |
| Legionella sp. | Metronidazole | 100 | 1 | 0 | 0 | 0 | 0 |
| Clostridium tetani | Ampicillin | 100 | 1 | 0 | 0 | 0 | 0 |
| Clostridium tetani | Ciprofloxacin | 0 | 0 | 100 | 1 | 0 | 0 |
| Prevotella melaninogenica | Amoxicillin oral (uncomplicated UTI only)1 | 100 | 1 | 0 | 0 | 0 | 0 |
| Prevotella melaninogenica | Nalidixic acid | 100 | 1 | 0 | 0 | 0 | 0 |
| Fusobacterium mortiferum | Nalidixic acid | 100 | 1 | 0 | 0 | 0 | 0 |
| Moraxella catarrhalis | Amikacin | 0 | 0 | 100 | 1 | 0 | 0 |
| Moraxella catarrhalis | Ceftazidime | 100 | 1 | 0 | 0 | 0 | 0 |
| Prevotella melaninogenica 1 | Benzylpenicillin | 100 | 1 | 0 | 0 | 0 | 0 |
| Veillonella parvula | Ertapenem | 100 | 1 | 0 | 0 | 0 | 0 |

**Table S2. Ward-specific distribution of antimicrobial-resistant bacteria**

| Ward name | Resistant antibiotic(s) | Resistant bacteria | N | % |
| --- | --- | --- | --- | --- |
| Maternity | Ceftriaxone (indications other than meningitis) | Klebsiella sp. | 1 | 0.19 |
| CCU | Ceftazidime | Escherichia coli | 1 | 0.19 |
| CCU | Ciprofloxacin | Escherichia coli | 1 | 0.19 |
| CCU | Nalidixic acid (U) | Escherichia coli | 1 | 0.19 |
| CCU | Ceftriaxone | Escherichia coli | 1 | 0.19 |
| CCU | Ceftriaxone (indications other than meningitis) | Enterococcus sp. | 1 | 0.19 |
| CCU | Cefotaxime | Escherichia coli | 1 | 0.19 |
| CCU | Cefotaxime | Klebsiella sp. | 2 | 0.38 |
| CCU | Ceftazidime | Escherichia coli | 3 | 0.57 |
| CCU | Ceftazidime | Klebsiella sp. | 1 | 0.19 |
| CCU | Ciprofloxacin | Escherichia coli | 3 | 0.57 |
| CCU | Ciprofloxacin | Klebsiella sp. | 2 | 0.38 |
| CCU | Nalidixic acid (U) | Escherichia coli | 3 | 0.57 |
| CCU | Nalidixic acid (U) | Klebsiella sp. | 2 | 0.38 |
| CCU | Trimethoprim-sulfamethoxazole | Escherichia coli | 2 | 0.38 |
| CCU | Trimethoprim-sulfamethoxazole | Klebsiella sp. | 2 | 0.38 |
| CCU | Nitrofurantoin (U) | Klebsiella sp. | 2 | 0.38 |
| CCU | Ceftriaxone | Escherichia coli | 1 | 0.19 |
| CCU | Ceftriaxone (indications other than meningitis) | Staphylococcus sp. | 1 | 0.19 |
| ICU | Ceftazidime | Escherichia coli | 1 | 0.19 |
| ICU | Nalidixic acid | Escherichia coli | 1 | 0.19 |
| ICU | Ampicillin | Streptococcus sp. | 1 | 0.19 |
| ICU | Cefotaxime | Escherichia coli | 1 | 0.19 |
| ICU | Ceftazidime | Escherichia coli | 3 | 0.57 |
| ICU | Ceftazidime | Proteus mirabilis | 1 | 0.19 |
| ICU | Tetracycline | Streptococcus sp. | 1 | 0.19 |
| ICU | Ciprofloxacin | Streptococcus sp. | 1 | 0.19 |
| ICU | Ciprofloxacin | Escherichia coli | 3 | 0.57 |
| ICU | Ciprofloxacin | Proteus mirabilis | 1 | 0.19 |
| ICU | Ciprofloxacin | Pseudomonas aeruginosa | 1 | 0.19 |
| ICU | Nalidixic acid (U) | Escherichia coli | 2 | 0.38 |
| ICU | Nalidixic acid (U) | Proteus mirabilis | 1 | 0.19 |
| ICU | Nalidixic acid (U) | Pseudomonas aeruginosa | 1 | 0.19 |
| ICU | Trimethoprim-sulfamethoxazole | Escherichia coli | 4 | 0.76 |
| ICU | Trimethoprim-sulfamethoxazole | Proteus mirabilis | 1 | 0.19 |
| ICU | Nitrofurantoin (U) | Escherichia coli | 1 | 0.19 |
| ICU | Nitrofurantoin (U) | Proteus mirabilis | 1 | 0.19 |
| ICU | Nitrofurantoin (U) | Pseudomonas aeruginosa | 1 | 0.19 |
| ICU | Ceftriaxone | Escherichia coli | 2 | 0.38 |
| ICU | Ceftriaxone | Proteus mirabilis | 1 | 0.19 |
| ICU | Ceftriaxone | Pseudomonas aeruginosa | 1 | 0.19 |
| ICU | Erythromycin | Streptococcus sp. | 1 | 0.19 |
| ICU | Clindamycin | Streptococcus sp. | 1 | 0.19 |
| ICU | Amoxicillin oral (uncomplicated UTI only)1 | Prevotella melaninogenica | 1 | 0.19 |
| ICU | Amoxicillin-clavulanic acid iv1 | Streptococcus pyogenes | 2 | 0.38 |
| ICU | Mecillinam oral (pivmecillinam) (uncomplicated UTI only), E. coli, Citrobacter spp., Klebsiella spp., Raoultella spp., Enterobacter spp. and P. mirabilis | Legionella sp. | 1 | 0.19 |
| ICU | Cefixime (uncomplicated UTI only) | Streptococcus pyogenes | 1 | 0.19 |
| ICU | Gentamicin | Legionella sp. | 1 | 0.19 |
| ICU | Nitrofurantoin | Legionella sp. | 1 | 0.19 |
| ICU | Ampicillin | Clostridium tetani | 1 | 0.19 |
| ICU | Nalidixic acid | Citrobacter freundii | 1 | 0.19 |
| ICU | Nalidixic acid | Prevotella melaninogenica | 1 | 0.19 |
| ICU | Ertapenem | Veillonella parvula | 1 | 0.19 |
| ICU | Metronidazole | Legionella sp. | 1 | 0.19 |
| Children | Ceftriaxone (indications other than meningitis) | Escherichia coli | 1 | 0.19 |
| Children | Ceftriaxone (indications other than meningitis) | Klebsiella sp. | 3 | 0.57 |
| Children | Trimethoprim-sulfamethoxazole | Escherichia coli | 1 | 0.19 |
| Children | Ceftriaxone (indications other than meningitis) | Staphylococcus sp. | 1 | 0.19 |
| Children | Ampicillin | Streptococcus sp. | 1 | 0.19 |
| Children | Cefotaxime | Klebsiella sp. | 2 | 0.38 |
| Children | Ceftazidime | Klebsiella sp. | 5 | 0.95 |
| Children | Ciprofloxacin | Streptococcus sp. | 1 | 0.19 |
| Children | Ciprofloxacin | Klebsiella sp. | 4 | 0.76 |
| Children | Nalidixic acid (U) | Escherichia coli | 1 | 0.19 |
| Children | Nalidixic acid (U) | Klebsiella sp. | 2 | 0.38 |
| Children | Trimethoprim-sulfamethoxazole | Streptococcus sp. | 1 | 0.19 |
| Children | Trimethoprim-sulfamethoxazole | Escherichia coli | 1 | 0.19 |
| Children | Trimethoprim-sulfamethoxazole | Klebsiella sp. | 6 | 1.14 |
| Children | Nitrofurantoin (U) | Klebsiella sp. | 3 | 0.57 |
| Children | Ceftriaxone | Klebsiella sp. | 2 | 0.38 |
| Children | Clindamycin | Streptococcus sp. | 1 | 0.19 |
| emergency | Cefotaxime (indications other than meningitis) | Escherichia coli | 1 | 0.19 |
| emergency | Ceftazidime | Escherichia coli | 3 | 0.57 |
| emergency | Ceftazidime | Klebsiella sp. | 3 | 0.57 |
| emergency | Ceftriaxone (indications other than meningitis) | Escherichia sp. | 1 | 0.19 |
| emergency | Ceftriaxone (indications other than meningitis) | Escherichia coli | 1 | 0.19 |
| emergency | Ceftriaxone (indications other than meningitis) | Escherichia coli ,enteroinvasive (EIEC) | 1 | 0.19 |
| emergency | Ceftriaxone (indications other than meningitis) | Klebsiella sp. | 1 | 0.19 |
| emergency | Gentamicin | Escherichia coli | 1 | 0.19 |
| emergency | Trimethoprim-sulfamethoxazole | Enterococcus sp. | 1 | 0.19 |
| emergency | Trimethoprim-sulfamethoxazole | Klebsiella sp. | 1 | 0.19 |
| emergency | Nalidixic acid | Escherichia coli | 1 | 0.19 |
| emergency | Cefotaxime | Streptococcus sp. | 1 | 0.19 |
| emergency | Cefotaxime | Staphylococcus sp. | 1 | 0.19 |
| emergency | Cefotaxime | Escherichia coli | 1 | 0.19 |
| emergency | Ceftazidime | Escherichia coli | 2 | 0.38 |
| emergency | Ceftazidime | Klebsiella sp. | 2 | 0.38 |
| emergency | Tetracycline | Streptococcus sp. | 1 | 0.19 |
| emergency | Tetracycline | Staphylococcus sp. | 1 | 0.19 |
| emergency | Ciprofloxacin | Streptococcus sp. | 1 | 0.19 |
| emergency | Ciprofloxacin | Escherichia coli | 7 | 1.33 |
| emergency | Ciprofloxacin | Klebsiella sp. | 3 | 0.57 |
| emergency | Nalidixic acid (U) | Enterococcus sp. | 1 | 0.19 |
| emergency | Nalidixic acid (U) | Escherichia coli | 7 | 1.33 |
| emergency | Nalidixic acid (U) | Klebsiella sp. | 4 | 0.76 |
| emergency | Trimethoprim-sulfamethoxazole | Enterococcus sp. | 1 | 0.19 |
| emergency | Trimethoprim-sulfamethoxazole | Staphylococcus sp. | 1 | 0.19 |
| emergency | Trimethoprim-sulfamethoxazole | Staphylococcus saprophyticus subsp. saprophyticus | 1 | 0.19 |
| emergency | Trimethoprim-sulfamethoxazole | Escherichia coli | 5 | 0.95 |
| emergency | Nitrofurantoin (U) | Escherichia coli | 1 | 0.19 |
| emergency | Nitrofurantoin (U) | Klebsiella sp. | 1 | 0.19 |
| emergency | Trimethoprim-sulfamethoxazole | Escherichia coli | 1 | 0.19 |
| emergency | Trimethoprim-sulfamethoxazole | Klebsiella sp. | 1 | 0.19 |
| emergency | Piperacillin | Streptococcus sp. | 1 | 0.19 |
| emergency | Ceftriaxone | Enterococcus sp. | 1 | 0.19 |
| emergency | Ceftriaxone | Staphylococcus sp. | 1 | 0.19 |
| emergency | Ceftriaxone | Escherichia coli | 4 | 0.76 |
| emergency | Ceftriaxone | Klebsiella sp. | 2 | 0.38 |
| emergency | Erythromycin | Streptococcus sp. | 1 | 0.19 |
| emergency | Erythromycin | Staphylococcus sp. | 2 | 0.38 |
| emergency | Erythromycin | Staphylococcus saprophyticus subsp. saprophyticus | 1 | 0.19 |
| emergency | Clindamycin | Streptococcus sp. | 1 | 0.19 |
| emergency | Clindamycin | Staphylococcus sp. | 2 | 0.38 |
| emergency | Ticarcillin-clavulanic acid | γ-Streptococcus | 1 | 0.19 |
| emergency | Gentamicin | γ-Streptococcus | 1 | 0.19 |
| emergency | Benzylpenicillin | Prevotella melaninogenica 1 | 1 | 0.19 |
| emergency | Nalidixic acid | Fusobacterium mortiferum | 1 | 0.19 |
| emergency | Ceftaroline | Klebsiella sp. | 1 | 0.19 |
| emergency | Nitrofurantoin (U) | Klebsiella sp. | 1 | 0.19 |
| emergency | Ceftriaxone (indications other than meningitis) | Klebsiella sp. | 1 | 0.19 |
| Laboratory | Ceftazidime | Escherichia coli | 2 | 0.38 |
| Laboratory | Ceftazidime | Klebsiella sp. | 1 | 0.19 |
| Laboratory | Gentamicin | Escherichia coli | 1 | 0.19 |
| Laboratory | Gentamicin | Klebsiella sp. | 1 | 0.19 |
| Laboratory | Gentamicin | Klebsiella sp. | 1 | 0.19 |
| Laboratory | Erythromycin | Proteus sp. | 1 | 0.19 |
| Laboratory | Cefotaxime (indications other than meningitis) | Klebsiella sp. | 1 | 0.19 |
| Laboratory | Ceftazidime | Escherichia coli | 8 | 1.52 |
| Laboratory | Ceftazidime | Klebsiella sp. | 6 | 1.14 |
| Laboratory | Ceftriaxone (indications other than meningitis) | Escherichia coli | 8 | 1.52 |
| Laboratory | Ceftriaxone (indications other than meningitis) | Klebsiella sp. | 8 | 1.52 |
| Laboratory | Nitrofurantoin | Klebsiella sp. | 1 | 0.19 |
| Laboratory | Nitroxoline E. coli | Escherichia coli | 1 | 0.19 |
| Laboratory | Trimethoprim-sulfamethoxazole | Streptococcus sp. | 1 | 0.19 |
| Laboratory | Trimethoprim-sulfamethoxazole | Klebsiella sp. | 1 | 0.19 |
| Laboratory | Erythromycin | Streptococcus sp. | 1 | 0.19 |
| Laboratory | Clindamycin | Streptococcus sp. | 2 | 0.38 |
| Laboratory | Clindamycin | Staphylococcus sp. | 1 | 0.19 |
| Laboratory | Erythromycin | Staphylococcus saprophyticus subsp. saprophyticus | 1 | 0.19 |
| Laboratory | Cefotaxime | Staphylococcus saprophyticus subsp. saprophyticus | 1 | 0.19 |
| Laboratory | Cefotaxime | Escherichia coli | 9 | 1.71 |
| Laboratory | Cefotaxime | Klebsiella sp. | 3 | 0.57 |
| Laboratory | Cefotaxime | Klebsiella pneumoniae subsp. pneumoniae | 2 | 0.38 |
| Laboratory | Ceftazidime | Escherichia coli | 5 | 0.95 |
| Laboratory | Ceftazidime | Klebsiella sp. | 4 | 0.76 |
| Laboratory | Ceftazidime | Klebsiella pneumoniae subsp. pneumoniae | 1 | 0.19 |
| Laboratory | Ceftazidime | Pseudomonas aeruginosa | 1 | 0.19 |
| Laboratory | Tetracycline | Escherichia coli | 1 | 0.19 |
| Laboratory | Ciprofloxacin | Streptococcus sp. | 1 | 0.19 |
| Laboratory | Ciprofloxacin | Staphylococcus sp. | 1 | 0.19 |
| Laboratory | Ciprofloxacin | Staphylococcus epidermidis | 1 | 0.19 |
| Laboratory | Ciprofloxacin | Escherichia coli | 20 | 3.8 |
| Laboratory | Ciprofloxacin | Klebsiella sp. | 8 | 1.52 |
| Laboratory | Ciprofloxacin | Klebsiella pneumoniae subsp. pneumoniae | 1 | 0.19 |
| Laboratory | Ciprofloxacin | Pseudomonas aeruginosa | 1 | 0.19 |
| Laboratory | Nalidixic acid (U) | Escherichia coli | 21 | 3.98 |
| Laboratory | Nalidixic acid (U) | Klebsiella sp. | 8 | 1.52 |
| Laboratory | Nalidixic acid (U) | Pseudomonas aeruginosa | 1 | 0.19 |
| Laboratory | Trimethoprim-sulfamethoxazole | Streptococcus sp. | 1 | 0.19 |
| Laboratory | Trimethoprim-sulfamethoxazole | Staphylococcus epidermidis | 1 | 0.19 |
| Laboratory | Trimethoprim-sulfamethoxazole | Staphylococcus saprophyticus subsp. saprophyticus | 1 | 0.19 |
| Laboratory | Trimethoprim-sulfamethoxazole | Escherichia coli | 15 | 2.85 |
| Laboratory | Trimethoprim-sulfamethoxazole | Klebsiella sp. | 5 | 0.95 |
| Laboratory | Trimethoprim-sulfamethoxazole | Klebsiella pneumoniae subsp. pneumoniae | 1 | 0.19 |
| Laboratory | Trimethoprim-sulfamethoxazole | Pseudomonas aeruginosa | 1 | 0.19 |
| Laboratory | Nitrofurantoin (U) | Staphylococcus sp. | 1 | 0.19 |
| Laboratory | Nitrofurantoin (U) | Escherichia coli | 8 | 1.52 |
| Laboratory | Nitrofurantoin (U) | Klebsiella sp. | 4 | 0.76 |
| Laboratory | Nitrofurantoin (U) | Klebsiella pneumoniae subsp. pneumoniae | 2 | 0.38 |
| Laboratory | Nitrofurantoin (U) | Pseudomonas aeruginosa | 1 | 0.19 |
| Laboratory | Ampicillin | Staphylococcus epidermidis | 1 | 0.19 |
| Laboratory | Ciprofloxacin | Klebsiella sp. | 1 | 0.19 |
| Laboratory | Trimethoprim-sulfamethoxazole | Escherichia coli | 1 | 0.19 |
| Laboratory | Ceftriaxone | Escherichia coli | 3 | 0.57 |
| Laboratory | Ceftriaxone | Pseudomonas aeruginosa | 1 | 0.19 |
| Laboratory | Erythromycin | Streptococcus sp. | 1 | 0.19 |
| Laboratory | Erythromycin | Staphylococcus sp. | 1 | 0.19 |
| Laboratory | Erythromycin | Staphylococcus epidermidis | 2 | 0.38 |
| Laboratory | Erythromycin | Staphylococcus saprophyticus subsp. saprophyticus | 1 | 0.19 |
| Laboratory | Erythromycin | Escherichia coli | 1 | 0.19 |
| Laboratory | Clindamycin | Streptococcus sp. | 1 | 0.19 |
| Laboratory | Clindamycin | Staphylococcus epidermidis | 1 | 0.19 |
| Laboratory | Clindamycin | Staphylococcus saprophyticus subsp. saprophyticus | 2 | 0.38 |
| Laboratory | Clindamycin | Escherichia coli | 1 | 0.19 |
| inpatient | Cefotaxime (indications other than meningitis) | No Growth | 1 | 0.19 |
| inpatient | Ceftazidime | Escherichia coli | 1 | 0.19 |
| inpatient | Ceftazidime | Klebsiella sp. | 2 | 0.38 |
| inpatient | Gentamicin | No Growth | 1 | 0.19 |
| inpatient | Ceftazidime | Escherichia coli | 2 | 0.38 |
| inpatient | Ceftazidime | Klebsiella sp. | 2 | 0.38 |
| inpatient | Ceftriaxone (indications other than meningitis) | Klebsiella sp. | 1 | 0.19 |
| inpatient | Ceftazidime | Escherichia coli | 1 | 0.19 |
| inpatient | Ceftriaxone (indications other than meningitis) | Escherichia coli | 1 | 0.19 |
| inpatient | Cefazolin | Klebsiella sp. | 1 | 0.19 |
| inpatient | Cefotaxime | Escherichia coli | 3 | 0.57 |
| inpatient | Cefotaxime | Klebsiella sp. | 2 | 0.38 |
| inpatient | Ceftazidime | Escherichia coli | 3 | 0.57 |
| inpatient | Ceftazidime | Klebsiella sp. | 2 | 0.38 |
| inpatient | Ciprofloxacin | Escherichia coli | 3 | 0.57 |
| inpatient | Ciprofloxacin | Klebsiella sp. | 8 | 1.52 |
| inpatient | Nalidixic acid (U) | Escherichia coli | 4 | 0.76 |
| inpatient | Nalidixic acid (U) | Klebsiella sp. | 7 | 1.33 |
| inpatient | Trimethoprim-sulfamethoxazole | Escherichia coli | 2 | 0.38 |
| inpatient | Trimethoprim-sulfamethoxazole | Klebsiella sp. | 6 | 1.14 |
| inpatient | Nitrofurantoin (U) | Escherichia coli | 3 | 0.57 |
| inpatient | Nitrofurantoin (U) | Klebsiella sp. | 3 | 0.57 |
| inpatient | Ceftriaxone | Escherichia coli | 1 | 0.19 |
| inpatient | Ceftriaxone | Klebsiella sp. | 3 | 0.57 |
| inpatient | Ceftriaxone (indications other than meningitis) | Klebsiella sp. | 1 | 0.19 |
| Woman surgery | Ampicillin iv1 | Streptococcus sp. | 1 | 0.19 |
| Woman surgery | Ceftazidime | Escherichia coli | 3 | 0.57 |
| Woman surgery | Ceftriaxone (indications other than meningitis) | Escherichia coli | 2 | 0.38 |
| Woman surgery | Ceftriaxone (indications other than meningitis) | Streptococcus constellatus (subsp. constellatus, pharyngis) | 1 | 0.19 |
| Woman surgery | Gentamicin | Escherichia coli | 1 | 0.19 |
| Woman surgery | Cefotaxime | Klebsiella sp. | 2 | 0.38 |
| Woman surgery | Ceftazidime | Escherichia coli | 3 | 0.57 |
| Woman surgery | Ceftazidime | Klebsiella sp. | 3 | 0.57 |
| Woman surgery | Ciprofloxacin | Escherichia coli | 5 | 0.95 |
| Woman surgery | Ciprofloxacin | Klebsiella sp. | 4 | 0.76 |
| Woman surgery | Nalidixic acid (U) | Escherichia coli | 5 | 0.95 |
| Woman surgery | Nalidixic acid (U) | Klebsiella sp. | 4 | 0.76 |
| Woman surgery | Trimethoprim-sulfamethoxazole | Escherichia coli | 4 | 0.76 |
| Woman surgery | Trimethoprim-sulfamethoxazole | Klebsiella sp. | 4 | 0.76 |
| Woman surgery | Nitrofurantoin (U) | Escherichia coli | 4 | 0.76 |
| Woman surgery | Nitrofurantoin (U) | Klebsiella sp. | 2 | 0.38 |
| Woman surgery | Trimethoprim-sulfamethoxazole | Klebsiella sp. | 1 | 0.19 |
| Woman surgery | Ceftriaxone | Escherichia coli | 4 | 0.76 |
| Woman surgery | Ceftriaxone | Klebsiella sp. | 2 | 0.38 |
| Woman surgery | Ampicillin iv1 | Group C β-Streptococcus | 1 | 0.19 |
| Woman surgery | Ampicillin oral (uncomplicated UTI only)1 | Group C β-Streptococcus | 1 | 0.19 |
| Woman surgery | Ampicillin-sulbactam iv1 | Group C β-Streptococcus | 1 | 0.19 |
| Woman surgery | Amoxicillin iv1 | Group C β-Streptococcus | 1 | 0.19 |
| Woman surgery | Ceftibuten (infections originating from the urinary tract) | Group C β-Streptococcus | 1 | 0.19 |
| Woman surgery | Gentamicin | Group C β-Streptococcus | 1 | 0.19 |
| Woman surgery | Minocycline | Enterococcus durans | 1 | 0.19 |
| Woman surgery | Doripenem | Group C β-Streptococcus | 1 | 0.19 |
| Woman surgery | Ceftazidime | Moraxella catarrhalis | 1 | 0.19 |
| Woman surgery | Azithromycin | Group C β-Streptococcus | 1 | 0.19 |
| Woman surgery | Doxycycline | Enterococcus durans | 1 | 0.19 |
| Woman surgery | Ceftazidime | Escherichia coli | 1 | 0.19 |
| man surgery | Ceftazidime | Pseudomonas aeruginosa | 1 | 0.19 |
| man surgery | Ciprofloxacin | Pseudomonas aeruginosa | 1 | 0.19 |
| man surgery | Nalidixic acid (U) | Pseudomonas aeruginosa | 1 | 0.19 |
| man surgery | Trimethoprim-sulfamethoxazole | Pseudomonas aeruginosa | 1 | 0.19 |
| man surgery | Nitrofurantoin (U) | Pseudomonas aeruginosa | 1 | 0.19 |
| man surgery | Ceftriaxone | Pseudomonas aeruginosa | 1 | 0.19 |
